# The impact of perfumes and cosmetic products on human health: a narrative review

**DOI:** 10.3389/ftox.2025.1646075

**Published:** 2025-08-29

**Authors:** Sharifa Alblooshi

**Affiliations:** College of Natural and Health Sciences, Zayed University, Dubai, United Arab Emirates

**Keywords:** Perfumes, cosmetics, health risks, synthetic chemicals, regulatory guidelines, endocrine disruption, heavy metals, consumer safety

## Abstract

**Background:**

The use of perfumes and cosmetic products is widespread, serving personal hygiene, aesthetic, and olfactory functions. However, concerns have been raised regarding the potential health impacts associated with long-term exposure to various ingredients used in these products.

**Objectives:**

This narrative review aims to synthesize evidence on the health risks associated with perfumes and cosmetics, focusing on specific health concerns, including fertility, respiratory health, cancer risk, allergies, skin disorders, endocrine disruption, and neurological effects. It also discusses the presence of heavy metals in cosmetics, regulatory challenges, and the need for transparency in ingredient disclosure.

**Methods:**

A comprehensive review of literature published between 2005 and 2025 was conducted, examining findings from interdisciplinary studies relevant to the health impacts of cosmetic and fragrance products. The review highlights health concerns linked to specific chemical components, including synthetic chemicals such as phthalates, parabens, and volatile organic compounds (VOCs).

**Results:**

The findings indicate that many synthetic chemicals in perfumes and cosmetics are associated with adverse health outcomes. These include allergies, respiratory issues, endocrine disruption, reproductive problems, and potentially cancer. Heavy metals in cosmetics also pose significant health risks. Despite regulatory guidelines, the cumulative and long-term effects of combined exposure to multiple cosmetic ingredients remain poorly understood and inadequately addressed.

**Conclusion:**

There is a pressing need for stricter regulatory oversight and improved transparency in ingredient disclosure to safeguard consumer health. Further research is required to clarify the long-term health risks associated with the daily use of cosmetic products and to develop safer alternatives.

## 1 Introduction

Perfumes and cosmetic products have become an integral part of daily life, transcending cultural and demographic boundaries (Park and Hong, 2024a). These products, which include an extensive array of items such as perfumes, deodorants, lotions, shampoos, soaps, and makeup, are valued not only for their practical purposes—such as personal hygiene and skin protection—but also for their capacity to enhance aesthetic appeal and provide sensory pleasure (Srinivasulu et al., 2022). With a history dating back to ancient civilizations, where natural extracts and oils were used for beautification and ritualistic purposes, the use of these products has evolved dramatically. Today, the cosmetics industry stands as a global powerhouse, worth billions of dollars and driven by relentless innovation and consumer demand (McMullen and Dell’Acqua, 2023).

The modern cosmetics industry is characterized by the use of sophisticated formulations that combine natural and synthetic ingredients. These innovations aim to achieve properties such as long-lasting fragrance, enhanced texture, vibrant color, and improved shelf life (Rico et al., 2024). While these advancements have undoubtedly elevated consumer experiences, they have also brought about a host of concerns regarding the potential health impacts of prolonged exposure to the chemical components of these products. Ingredients such as phthalates, parabens, volatile organic compounds (VOCs), and heavy metals are commonly used to achieve desired cosmetic effects, but growing evidence suggests that they may pose significant health risks.

Research in recent decades has linked certain cosmetic ingredients to a range of adverse health outcomes, including skin irritation, respiratory issues, allergic reactions, endocrine disruption, reproductive problems, and even an elevated risk of certain cancers. For instance, phthalates and parabens, often used as preservatives and stabilizers, have been associated with hormone mimicry, which can interfere with endocrine functions (Ahmed et al., 2024). Similarly, VOCs—emitted as gases from fragrances and aerosol products—have been implicated in respiratory disorders and neurological effects. The presence of heavy metals such as lead, cadmium, and mercury in certain cosmetics further exacerbates health concerns, as these elements are known to accumulate in the body and exert toxic effects over time (Witkowska et al., 2021).

Despite the potential health implications, the regulatory landscape governing cosmetics and personal care products remains fragmented and, in some cases, insufficiently stringent. While agencies such as the U.S. Food and Drug Administration (FDA) and the European Medicines Agency (EMA) have implemented guidelines to ensure product safety, these regulations often fall short in addressing the cumulative and long-term effects of repeated exposure to multiple chemical ingredients (Bopp et al., 2018). Additionally, the lack of transparency in ingredient disclosure—with many formulations protected under the guise of trade secrets—complicates efforts to assess and mitigate risks effectively. This regulatory gap has sparked growing public concern and calls for stricter oversight to safeguard consumer health (Iqbal and Biller-Andorno, 2022). The average consumer is exposed to a multitude of personal care products daily, ranging from toothpaste and shampoo in the morning to moisturizers and perfumes throughout the day. This habitual use results in compounded exposure to a cocktail of chemicals, the interactions and combined effects of which remain poorly understood (Elcombe et al., 2022). Furthermore, vulnerable populations such as pregnant women, children, and individuals with pre-existing health conditions may be at heightened risk, underscoring the need for targeted research and tailored safety recommendations.

Existing reviews on the safety of perfumes and cosmetics have primarily focused on specific aspects, such as dermatological effects or toxicological impacts, often leaving critical gaps in the broader understanding of their implications for human health (Manful et al., 2024). This narrative review seeks to bridge these gaps by synthesizing evidence from diverse disciplines, including dermatology, toxicology, endocrinology, and environmental health (Nunes et al., 2024). By doing so, it aims to provide a holistic perspective on the health risks associated with the use of cosmetic products and fragrances (Alnuqaydan, 2024).

Specifically, this review will explore the impact of cosmetic ingredients on fertility, respiratory health, cancer risk, allergies, skin disorders, endocrine function, neurological well-being, and the presence of heavy metals. It will also examine emerging evidence on potential risks, offering insights into areas requiring further investigation. Ultimately, this review underscores the urgent need for more comprehensive regulatory frameworks, greater transparency in ingredient disclosure, and heightened consumer awareness to address the multifaceted health challenges posed by the widespread use of cosmetic and fragrance products.

Historically, discussions surrounding the safety of cosmetics have primarily focused on well-documented health issues, such as their effects on fertility, respiratory health, cancer risks, and allergic reactions (Tong et al., 2023). However, a growing body of research has begun to uncover a broader spectrum of potential health implications that extend beyond these commonly examined areas. This expanding field of inquiry emphasizes the need to explore other conditions that may arise due to the complex interactions between cosmetic ingredients and the human body (Alnuqaydan, 2024).

Among these emerging concerns are skin disorders, such as dermatitis, eczema, and allergic reactions, which affect a significant number of individuals following the use of certain cosmetic products. Additionally, there is increasing evidence that specific chemicals in cosmetics may disrupt endocrine function, potentially interfering with hormonal regulation and leading to adverse effects on reproductive and metabolic systems. Furthermore, some studies suggest a link between exposure to fragrances and neurological outcomes, including headaches, mood disturbances, and other cognitive effects (Li et al., 2024).

As the scope of research widens, it becomes essential to consider these additional conditions to gain a more comprehensive understanding of the potential health risks associated with cosmetics (Pistollato et al., 2021). This exploration underscores the importance of rigorous testing, informed consumer choices, and regulatory measures to ensure the safe use of these everyday products. The following sections delve into these emerging concerns in greater detail, highlighting the need for heightened awareness and ongoing scientific investigation (Pastor-Nieto and Gatica-Ortega, 2021). The main objectives are to assess the impacts of perfumes and cosmetics on fertility, lung health, cancer risk, allergies, and other conditions such as skin disorders, endocrine disruption, and neurological effects, and the effects of heavy metals.

## 2 Methodology

The methodology for this narrative review on the health impacts of perfumes and cosmetic products encompasses a literature collection based on inclusion and exclusion criteria, and qualitative synthesis of peer-reviewed studies. The goal is to consolidate findings that examine potential health risks associated with cosmetic and fragrance use, particularly focusing on fertility, lung health, cancer risk, allergies, skin disorders, endocrine disruption, neurological effects, and effects of heavy metals. To comprehensively analyze the health impacts of perfumes and cosmetic products, peer-reviewed studies were sourced from databases including PubMed, Google Scholar, and ResearchGate. Keywords such as “perfume toxicity,” “cosmetic ingredients health effects,” “fragrance VOCs,” “allergies and cosmetics,” “endocrine disruption cosmetics,” and “cancer risk cosmetics” were employed to ensure a wide scope of relevant literature. All articles used were peer-reviewed and sourced from reputable journals to ensure scientific rigor. Studies that were published in the years between 2005 and 2025 were included to provide a well rounded and comprehensive perspective on the topic.

### 2.1 Inclusion criteria

- Studies that examine the effects of perfumes and cosmetics on human health.
- Both observational and experimental research were included.
- Focus on fertility, lung health, cancer risk, allergies, skin disorders, endocrine disruption, neurological effects, and the effects of heavy metals in cosmetics.

### 2.2 Exclusion criteria

- Non-peer-reviewed articles.
- Opinion pieces and anecdotal evidence.
- Studies with limited relevance or non-generalizable results (e.g., small sample sizes).

## 3 Results and discussion

**FIGURE 1:**
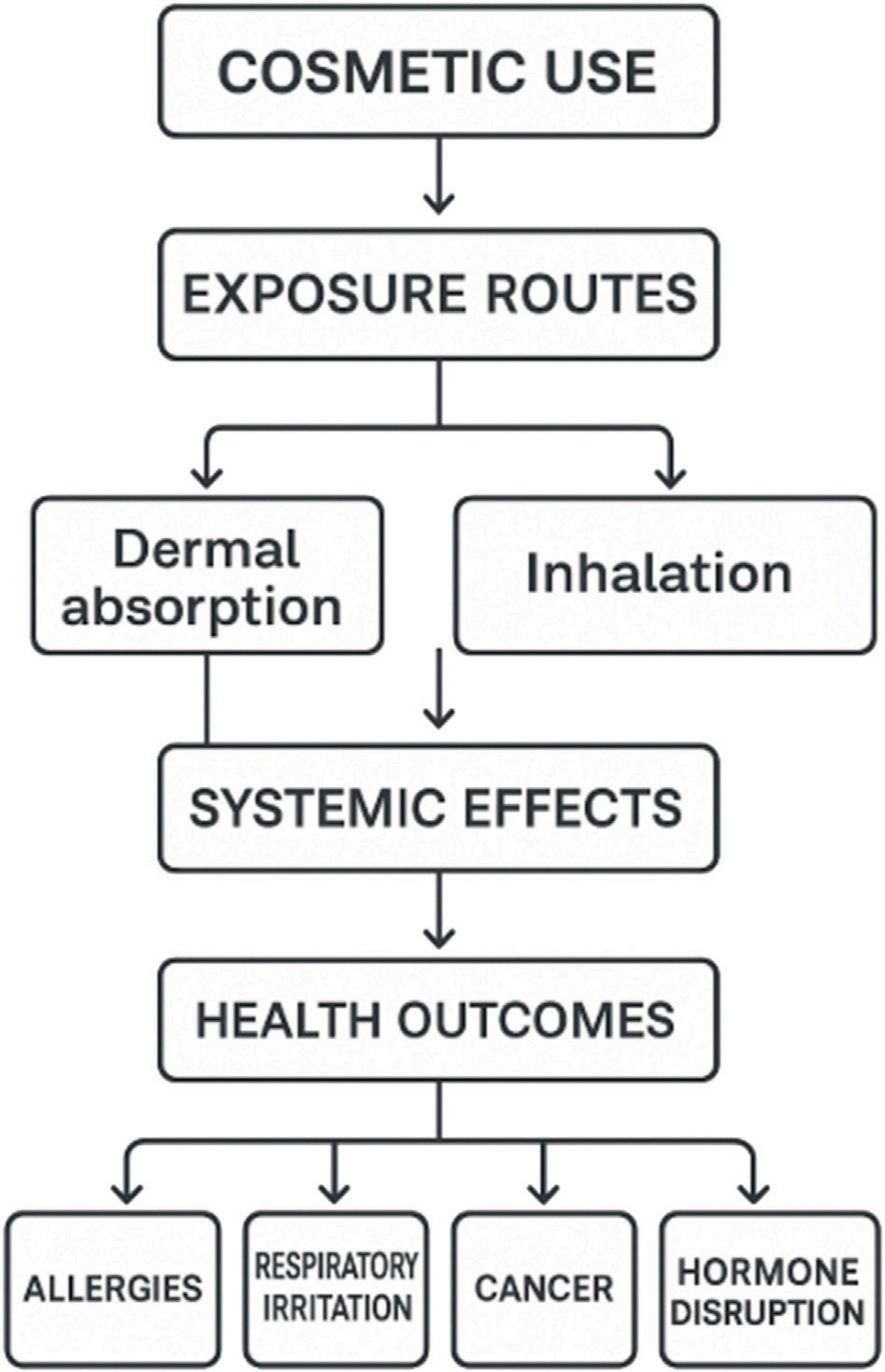
Health impact pathway of cosmetic product use.

Figure 1 illustrates the pathway through which cosmetic use can lead to adverse health outcomes, emphasizing the mechanisms of exposure and subsequent systemic effects. It begins with the application of cosmetic products, which introduces potential contaminants into the body via two primary exposure routes: dermal absorption and inhalation. Dermal absorption occurs when substances penetrate the skin, while inhalation involves breathing in volatile compounds, sprays, or powders. Both routes can result in systemic distribution of these chemicals throughout the body, potentially disrupting normal physiological functions. This systemic exposure may lead to various health outcomes, including allergic reactions, respiratory irritation, cancer, and hormonal disruption. The diagram highlights the importance of understanding not just the ingredients in cosmetic products but also how they interact with the body over time, particularly through repeated use, which can amplify cumulative exposure and health risks.

The use of cosmetics, while an integral component of personal care and beauty practices, introduces a multifaceted array of potential health risks stemming from their chemical compositions (Abiodun and Ayeleru, 2025). This discussion explores key areas of concern, highlighting evidence from diverse studies and emphasizing the need for increased public awareness, enhanced research efforts, and stringent regulatory measures to ensure consumer safety.

### 3.1 Endocrine disruption

Endocrine-disrupting chemicals (EDCs) such as parabens, phthalates, bisphenols, and other synthetic compounds frequently found in cosmetics pose significant threats to human health (Thompson, 2018). These compounds, prevalent in personal care products including perfumes, lotions, nail polishes, and hair sprays, have been shown to interfere with the endocrine system, thereby disrupting hormonal homeostasis (Anderson et al., 2022). A biomonitoring study involving 144 Norwegian adults found widespread urinary detection of parabens, bisphenols, phthalates, and UV filters, with exposure levels linked to hormone-related toxicity and metabolic disruption (Husøy et al., 2019). These substances can mimic, inhibit, or interfere with endogenous hormone signaling, thereby disrupting critical regulatory pathways associated with reproduction, metabolism, and thyroid function. EDCs can interfere with hormones such as estrogen, testosterone, thyroid hormones, and insulin, provoking physiological disturbances across multiple systems. See Table 1 for health risks associated with common cosmetic ingredients and contaminants across multiple bodily systems and functions.

**TABLE 1:** Common cosmetic contaminants: uses, exposure, health risks, regulations, and vulnerable populations.

Chemical contaminant	Typical uses in cosmetics	Average exposure/Levels	Potential health risks	Regulatory limits	Vulnerable populations	Ref
Parabens (e.g., methylparaben, propylparaben)	Preservatives	Up to 0.8%	Endocrine disruption, reproductive toxicity, potential breast cancer link	EU: max 0.8% per paraben; banned in leave-on products for children <3 years	Pregnant women, adolescents	Darbre and Harvey (2014), CDC et al. (2019), Vale et al., 2022; Suresh (2013)
Phthalates (e.g., DEP, DBP)	Solvent or fixative	Up to 1%	Reproductive toxicity, reduced sperm quality, neurodevelopmental effects	EU: Banned US: No restriction in US	Pregnant women, infants	Hauser and Calafat (2005), Mukherjee Das et al. (2022), CDC et al. (2019)
Formaldehyde and releasers (e.g., DMDM hydantoin)	Preservatives	0.2%–1.0%	Group 1 carcinogen (IARC), skin sensitization, asthma	EU: banned US: ≤0.2% in non-aerosol cosmetics	Hairdressers, children	De Groot and Flyvholm (2020), Smith and Hotchkiss (2023), IARC (2006)
Heavy Metals (e.g., lead, cadmium, arsenic)	Contaminant	Lead: 0.000719% Cadmium: 0.00036%	Neurotoxicity, carcinogenicity, developmental delay, nephrotoxicity	EU: banned unless technically unavoidable FDA: ≤10 ppm lead	Children, pregnant women	Limiting lead in lipstick and other cosmetics FDA, 2024; Nnorom et al. (2005), Attard et al. (2022)
Triclosan	Antibacterial	Up to 0.3%	Thyroid hormone disruption, antimicrobial resistance	EU: banned US: banned in hand soaps (2017)	Children, endocrine-sensitive individuals	Limiting lead in lipstick and other cosmetics FDA, 2024; Allmyr et al. (2006), Rees Clayton et al. (2010), FDA (2016)
UV Filters (e.g., oxybenzone/BP-3)	Sunscreen agent (absorbs UV radiation)	Up to 6%	Endocrine disruption, allergic reactions, coral reef toxicity	EU: up to 6% (oxybenzone) FDA: generally recognized as safe	Pregnant women, frequent sunscreen users	Janjua et al. (2007), Krause et al. (2012)
VOCs (e.g., Limonene, Toluene)	Fragrance ingredient	Varies	Respiratory irritation, neurotoxicity	No specific limits	Consumers with respiratory issues	Zhou et al. (2023)

Additionally, a substantial body of research has correlated EDC exposure with conditions such as obesity, diabetes, polycystic ovarian syndrome (PCOS), and infertility. Phthalates and bisphenols are particularly implicated in obesity through their impact on adipocyte differentiation, metabolic rate, and appetite regulation (Hauser and Calafat, 2005). These chemicals contribute to increased lipid accumulation and impaired appetite signaling, facilitating excessive weight gain. Phthalates have also been shown to impair insulin sensitivity and alter glucose metabolism, contributing to the pathogenesis of type 2 diabetes. Critically, the timing of exposure plays a pivotal role in determining health outcomes. Prenatal and early childhood periods represent windows of heightened vulnerability, during which EDCs can induce lasting developmental and physiological alterations. For example, bisphenol A (BPA), found in plastic packaging and some cosmetic containers, can cross the placental barrier and disrupt fetal endocrine function (Limiting lead in lipstick and other cosmetics FDA, 2024). Emerging evidence indicates that the effects of EDCs may extend across generations through epigenetic mechanisms. Maternal exposure to EDCs can induce modifications in gene expression that are heritable, even in the absence of continued exposure. These transgenerational effects may increase the risk of metabolic dysfunction, endocrine disorders, and reproductive abnormalities in descendants (Nnorom et al., 2005).

Given their widespread presence in personal care products, urgent regulatory action is warranted. While some jurisdictions have initiated restrictions on EDCs in cosmetics, enforcement remains fragmented, and many formulations still contain potentially hazardous chemicals (Allmyr et al., 2006). Regulatory frameworks must prioritize the establishment of concentration limits, pre-market safety assessments, and transparent labeling. Policies encouraging safer, non-toxic alternatives can help protect public health and reduce long-term exposure risks. Consumer engagement is also essential in mitigating EDC exposure. By selecting products free of parabens, phthalates, and bisphenols, individuals can reduce personal risk. Supporting brands that prioritize safety, and transparency encourages broader industry reform. The rising demand for clean, non-toxic cosmetics is already catalyzing a shift toward safer formulations and more sustainable manufacturing practices (Rees Clayton et al., 2010; Janjua et al., 2007). In summary, the endocrine-disrupting potential of numerous cosmetic ingredients necessitates comprehensive action from regulatory bodies, industry stakeholders, and consumers. A coordinated approach involving regulatory reform, scientific innovation, and public awareness is essential to reduce exposure and safeguard hormonal health across the lifespan.

### 3.2 Fertility

As discussed in Section 3.1, endocrine-disrupting chemicals such as phthalates and parabens impair hormonal regulation, leading to fertility issues. In females, these compounds can compromise ovarian function, oocyte quality, and hormonal signaling pathways necessary for conception and gestation. In males, EDCs can impair spermatogenesis, reduce sperm motility, and lower sperm counts, collectively undermining fertility. Additionally, exposure to these chemicals has been associated with early-onset puberty in both sexes, which may disrupt growth trajectories, sexual maturation, and long-term reproductive outcomes (De Groot and Flyvholm, 2020).

Mechanistically, phthalates disrupt the hypothalamic-pituitary-gonadal (HPG) axis by acting as exogenous ligands that can mimic, antagonize, or modulate the activity of endogenous hormones (Di Cicco et al., 2021). Disruption of the HPG axis alters the synthesis and secretion of key reproductive hormones, leading to documented fertility impairments (Gould et al., 2024). Epidemiological studies have linked phthalate exposure to reduced semen quality in men and menstrual disturbances in women (Elsaid and Ahmed, 2021). Importantly, their reproductive toxicity extends to developmental windows of heightened vulnerability, particularly during gestation. Prenatal exposure has been linked to adverse birth outcomes, such as cryptorchidism in male infants, suggesting interference with utero-androgen signaling pathways (Khalid and Abdollahi, 2021). Similarly, Octyl Methoxycinnamate (OMC), a UV filter commonly used in sunscreens and cosmetics, has been shown to affect fetal development. In an *in vivo* animal study, pregnant Wistar rats exposed to OMC had significantly reduced maternal thyroxine (T4) levels, and their male offspring displayed decreased testosterone levels, lower sperm counts, and reduced weights of reproductive organs such as the prostate and testes (Axelstad et al., 2011). These effects persisted into adulthood, though the study’s applicability is limited by the lack of human data. Furthermore, disruptions in the timing of pubertal onset, either precocious or delayed puberty, have been documented in populations with elevated early-life phthalate exposure, indicating that these chemicals may perturb developmental endocrine pathways with long-term consequences for reproductive health (Lazou, 2024).

Parabens, another class of EDCs, exert estrogenic effects by binding to estrogen receptors and mimicking the activity of endogenous estrogens (Akano et al., 2024). By exhibiting estrogenic activity, they can overstimulate estrogen receptors and disrupt endocrine homeostasis (Darbre and Harvey, 2014). This interaction has raised concerns about their potential role in impairing female fertility. *In vitro* studies show that butylparaben and propylparaben strongly mimicked estrogen, activating both ERα and ERβ, while ethylparaben had weaker effects, and methylparaben was inactive (Gomez et al., 2005). UV filters such as OMC, 4-Methylbenzylidene camphor (4-MBC), Octyl-Dimethyl PABA (OD-PABA), and Homosalate (HMS) selectively activated ERα, indicating a risk of tissue-specific endocrine disruption. Musk ketone and galaxolide weakly activated ERα; however, they raise concern as they persist in the environment and bioaccumulate. The study used a validated receptor-specific assay and benchmarking against 17β-estradiol, the primary endogenous estrogen. While this approach characterizes receptor binding and activation potential, it does not account for the metabolic transformation of these compounds within the human body, limiting its applicability to real-world exposure scenarios.

Addressing this gap, a single-blinded human exposure study involving thirty-two healthy adults (15 young men and 17 postmenopausal women) exposed participants to sunscreen formulations containing benzophenone-3 (BP-3), OMC, and 4-MBC (Janjua et al., 2004). Despite systemic absorption confirmed by detectable levels of these UV filters in both blood and urine samples, the study found no biologically significant alterations in reproductive hormones, including FSH, LH, estradiol, testosterone, and inhibin B. The study was limited in its short exposure period, relatively small sample size, and lack of long-term follow-up. It also did not evaluate cumulative exposure effects or possible delayed endocrine outcomes. Nonetheless, the detection of UV-filters in the blood raises concern, and safety thresholds should account for systemic bioavailability of these ingredients during repeated daily use.

Inverse associations have been observed between urinary paraben concentrations and ovarian reserve markers, including antral follicle count and anti-Müllerian hormone levels (Alnuqaydan, 2024). These suggest that parabens may impair reproductive potential by compromising oocyte quality, with implications for fertility and assisted reproductive outcomes. Genetic susceptibility may also mediate individual responses to EDCs. A biomonitoring study that looked at 195 healthy young men in Denmark, with and without filaggrin (*FLG*) gene mutations (a gene that affects the skin’s natural barrier function) found that in men with *FLG* mutations, higher urinary concentrations of bisphenol A (BPA) and UV filters such as BP-1, BP-3, and 4-HBP were associated with increased serum testosterone and estradiol levels, decreased follicle-stimulating hormone (FSH), and reduced sperm motility (Joensen et al., 2018). These effects were not observed in non-carriers, showing that endocrine response could be subject to the role of genetic predisposition. No significant associations were found between urinary paraben levels and hormone levels or semen health in either group. These findings underscore the importance of personalized risk assessment based on genetic susceptibility.

The reproductive risks associated with cosmetic EDCs are compounded by widespread and repeated exposure. Daily application of multiple personal care products results in cumulative exposure, contributing to systemic bioaccumulation over time (Marusic Kristina, 2023). This risk is especially concerning for susceptible subgroups, such as pregnant individuals, adolescents undergoing pubertal development, and those with pre-existing endocrine disorders, who may exhibit heightened biological sensitivity to these compounds. Given the persistence and biological activity of EDCs in cosmetics, a precautionary approach is warranted. Regulatory frameworks should enforce rigorous safety assessments of cosmetic ingredients, mandate clear labeling of EDC content, and incentivize the development of non-disruptive alternatives. Public health campaigns must also play a role in disseminating evidence-based guidance to help consumers make informed choices about personal care product use (Mukherjee Das et al., 2022). In summary, converging evidence from mechanistic, epidemiological, and developmental studies supports a causal link between chronic exposure to phthalates, parabens, and other EDCs in cosmetics and adverse reproductive health outcomes (Hager et al., 2022). These findings underscore the need for proactive policy interventions and consumer education to mitigate risks and safeguard reproductive potential across the lifespan and for future generations (Beyuo et al., 2024).

### 3.3 Lung health and asthma

The respiratory consequences of cosmetic product use, particularly those containing synthetic fragrances and aerosolized components, have emerged as a critical area of public health concern. Numerous products, such as perfumes, deodorants, hair sprays, and air fresheners, emit Volatile Organic Compounds (VOCs), which are recognized contributors to both indoor and outdoor air pollution (Srinivasulu et al., 2022). These VOCs not only act as primary airborne irritants but also undergo chemical reactions with other indoor pollutants, especially nitrogen oxides, to form secondary toxicants, including ozone and ultrafine particulate matter. This dynamic significantly elevates respiratory health risks, particularly in enclosed or poorly ventilated environments where pollutant concentrations can accumulate rapidly (Srinivasulu et al., 2022).

VOCs are a chemically diverse group of compounds frequently present in scented cosmetic products. They include terpenes (e.g., limonene), alcohols, aldehydes, and esters, many of which have known irritant or sensitizing properties (Dhiman et al., 2024). Inhalation of these compounds can provoke acute respiratory symptoms such as coughing, wheezing, and dyspnea by irritating the mucosal linings of the upper and lower respiratory tract (Singh et al., 2023). Of particular concern is their role in generating ground-level ozone when they interact with other ambient pollutants. Ozone is a well-established pulmonary irritant associated with airway inflammation, reduced lung function, and increased hospital admissions for respiratory conditions (Bird et al., 2021). Chronic exposure to elevated VOC and ozone levels has been implicated in the development and exacerbation of asthma, particularly among sensitive subpopulations.

Persistent exposure to aerosolized cosmetics, including spray deodorants, dry shampoos, and setting sprays is associated with structural changes in lung tissue, impaired gas exchange, and a heightened risk of respiratory infections. Vulnerable groups such as children whose lungs are still maturing and elderly individuals with pre-existing conditions like asthma or COPD are especially susceptible to these effects, with exposure linked to increased frequency and severity of exacerbations, hospital visits, and longterm morbidity (Attard et al., 2022; Ullah et al., 2023).

The implications of cosmetic-related respiratory distress extend beyond personal health, impacting healthcare systems and occupational environments. Increased incidence of asthma and other chronic respiratory diseases due to VOC and particulate exposure translates into greater demand for medical care, productivity losses, and elevated healthcare expenditures (Raza-Naqvi et al., 2022). Occupational exposure in high-risk settings, such as beauty salons, spas, and cosmetic manufacturing facilities, is particularly concerning due to the intensity and duration of exposure. Workers in these environments often experience prolonged inhalation of concentrated chemical mixtures, frequently without adequate personal protective equipment or ventilation systems, placing them at risk for chronic respiratory conditions and occupational asthma (Manful et al., 2024).

### 3.4 Cancers

Cosmetic products, while widely used for enhancing appearance and hygiene, can also expose users to harmful substances linked to carcinogenesis (Scopelliti et al., 2021). Mounting scientific evidence implicates several common cosmetic ingredients in cancer development, underscoring the need for stricter regulation, safer formulations, and informed consumer choices. Understanding these associations is crucial to public health protection (Lohmann, 2024).

#### 3.4.1 Hormone-mimicking chemicals: parabens and phthalates

Given their endocrine-disrupting properties (see Section 3.1), parabens and phthalates can also stimulate cell proliferation in estrogen-sensitive tissues, raising concern for hormone-related cancers such as breast and ovarian cancer (Lazzarini et al., 2018). Notably, even low-dose, chronic exposure to these chemicals has been associated with biological effects due to their capacity for bioaccumulation (Groot, 2021). Widespread use in items like deodorants, moisturizers, nail polish, and hair products ensures near-continuous human contact (Aparicio-Soto et al., 2022). In the absence of enforceable limits or mandatory disclosure, consumers are often unaware of their exposure. Implementing precautionary regulations and promoting the development of endocrine-safe alternatives is imperative to reduce carcinogenic risks.

#### 3.4.2 Talc and asbestos contamination

Talc, valued in cosmetics for its texture-enhancing and moisture-absorbing properties, has drawn concern due to potential asbestos contamination, a result of talc and asbestos often co-occurring geologically (Mawhirt and Fonacier, 2022). Asbestos fibers, when inhaled or used in the genital area, have been strongly associated with mesothelioma and ovarian cancer, respectively (Lejding, 2024).

Although regulatory agencies require asbestos-free talc, inconsistencies in enforcement and testing practices have left gaps in consumer protection (Tammaro et al., 2012). In particular, some regions lack mandatory, standardized testing for asbestos contamination. Strengthening quality assurance procedures and ensuring transparent ingredient sourcing are critical steps to safeguard public health. Safer talc alternatives and clear labeling would further mitigate exposure risks (Fonacier et al., 2022).

#### 3.4.3 Heavy metals

Heavy metals such as lead and arsenic, often present as unintentional contaminants in cosmetics, pose another significant carcinogenic threat (Line et al., 2023). Lead, found in some lipsticks, can accumulate in the body and is linked to DNA damage and endocrine disruption. Arsenic, detected in certain skin-lightening creams, is classified as a Group 1 carcinogen and associated with cancers of the skin, lungs, and bladder (Sicherer et al., 2022). A toxicological analysis of nine widely available fairness creams showed mercury and zinc at concentrations up to 141 mg/kg and 138 mg/kg, mercury levels exceeding international safety thresholds and being associated with elevated hazard quotients (HQ) and lifetime cancer risk (LCR) scores (Irfan et al., 2022). The products also contained lead, cadmium, nickel, and chromium, all linked to carcinogenic effects. These metals often enter cosmetic formulations through contaminated raw materials or inadequate quality control during manufacturing. Because of their ability to persist in tissues and their cumulative toxicity, even low-level, chronic exposure raises serious health concerns (Sicherer et al., 2022). To minimize risks, enhanced contaminant screening, supply chain monitoring, and regulatory oversight are urgently required. Educating the public about these hazards will further reduce unnecessary exposure.

A laboratory-based analysis of 95 cosmetic products reported that all samples contained multiple toxic metals, including arsenic, lead, cadmium, chromium, and nickel (Salles et al., 2023). Arsenic was the dominant contributor to lifetime cancer risk (LCR), especially among children exposed via ingestion (LCR up to 10^−5^) and adults via dermal contact (LCR up to 10^−3^). Further raising these concerns, laboratory-based toxicological risk assessment of 14 facial cosmetics revealed that several lipsticks and eye shadows contained lead, chromium, and mercury at concentrations far exceeding international safety limits. Lead levels reached 27.0 mg/kg in lipstick and 40.9 mg/kg in eye shadow, while chromium was as high as 149 mg/kg and mercury up to 138 mg/kg (Voica et al., 2023). These metals are associated with carcinogenicity. On the other hand, another large-scale laboratory risk assessment analyzed 200 cosmetic products from the Korean market and found that most heavy metals, including chromium, lead, cadmium, mercury, and arsenic, posed negligible cancer and systemic toxicity risks under normal use (Lim et al., 2018). Lifetime cancer risk (LCR) and hazard indices (HI) for all metals remained below safety thresholds. However, a subset of lip and eye products used by heavy consumers exceeded acceptable daily intake levels for Cr^6+^, Mn, and Pb, suggesting targeted concern for frequent users. These findings highlight the importance of product-specific use patterns and support routine monitoring to ensure safety across varying levels of cosmetic use.

#### 3.4.4 Broader implications and the path forward

The recurring presence of carcinogens in cosmetics highlights fundamental gaps in global regulatory frameworks (Aleksandra and Lelieveld, 2022). Unlike drugs, many cosmetic products bypass pre-market safety evaluations. This regulatory void allows ingredients with limited toxicological data to enter the market unchecked. A harmonized global approach involving ingredient testing, safety thresholds, and post-market surveillance is needed to ensure consistent consumer protection. Public health initiatives should prioritize awareness campaigns, transparent labeling, and investment in green chemistry solutions (El Maghraby and Arafa, 2023). Collaborative regulatory efforts across borders can help align standards and enforcement mechanisms, fostering a safer cosmetics landscape. Such actions are vital not only to reduce cancer incidence but also to promote accountability and sustainability in the personal care industry (El Maghraby and Arafa, 2023).

In summary, several commonly used cosmetic ingredients, parabens, phthalates, talc, and heavy metals, pose potential carcinogenic risks that warrant immediate attention (Amutha et al., 2024). A combined effort by regulators, manufacturers, and consumers is essential to transition toward safer formulations that protect long-term health. Reinforcing trust in cosmetic safety requires science-driven policy, rigorous oversight, and a shift toward transparency and prevention (Lohmann, 2024). Mitigating these risks requires a comprehensive public health and regulatory response. Public education initiatives are essential to inform consumers about the potential hazards posed by fragranced and aerosolized cosmetic products, especially for those with existing respiratory vulnerabilities. Improved labeling standards that indicate the presence and potential effects of VOCs and particulate matter are needed to facilitate informed consumer decision-making (Alurame Eruaga, 2024). Regulatory agencies should implement stricter limits on VOC emissions in cosmetic formulations and mandate routine safety testing for inhalation risks, particularly for spray products.

Advances in formulation science also offer pathways to reduce respiratory hazards. Reformulating products to use low- or zero-VOC ingredients, transitioning to solid or water-based cosmetic formats, and increasing the availability of unscented or naturally derived fragrance options can significantly lower exposure levels (Anagnosti et al., 2021). For occupational settings, enforcing indoor air quality standards, providing appropriate ventilation systems, and ensuring access to protective gear are critical strategies for protecting worker health (Smith and Hotchkiss, 2023).

In conclusion, while cosmetics are widely used and culturally embedded, their potential to harm respiratory health especially through VOCs and fine particulate emissions warrants significant attention (Lohmann, 2024). Individuals with asthma, COPD, or other respiratory conditions, along with children and the elderly, face heightened risks. Through targeted regulation, innovation in product development, and greater public awareness, meaningful progress can be made in reducing these exposures and protecting the population’s lung health (Lohmann, 2024).

### 3.5 Allergies

Allergic reactions to cosmetics, particularly allergic contact dermatitis (ACD), represent a significant proportion of adverse events associated with personal care products (Lin et al., 2024). These hypersensitivity reactions are typically triggered by specific chemical constituents such as fragrances, formaldehyde-releasing preservatives, and synthetic dyes. These allergens can provoke a range of responses from localized inflammation to, in rare cases, systemic effects like urticaria and anaphylaxis (Novak-Bilić et al., 2018).

#### 3.5.1 Pathophysiology of cosmetic-induced allergies

ACD results primarily from type IV delayed-type hypersensitivity, mediated by T lymphocytes. After initial exposure, small allergenic molecules (haptens) penetrate the skin barrier and bind to carrier proteins, forming immunogenic complexes (Khan and Alam, 2019). These are processed by Langerhans cells, which migrate to lymph nodes to activate T cells. Upon re-exposure, memory T cells initiate a robust inflammatory cascade, resulting in the clinical features of dermatitis (Maden, 2024). In contrast, irritant contact dermatitis (ICD) arises from cumulative damage to the skin barrier by repeated exposure to irritants. Although not immune-mediated, ICD can exacerbate or coexist with ACD, complicating diagnosis and treatment. Accurate differentiation is essential, as management strategies vary (Parkes, 2023).

#### 3.5.2 Common allergenic components

- Fragrances: Among the most prevalent sensitizers, fragrance ingredients like cinnamic aldehyde, eugenol, and isoeugenol are frequent triggers of ACD. Cross-reactivity is common, particularly in individuals allergic to balsam of Peru, which shares chemical constituents with many fragrance compounds (Martini, 2022).
- Preservatives: Widely used to prevent microbial contamination, preservatives such as formaldehyde-releasing agents, parabens, and isothiazolinones pose notable allergenic risks. Formaldehyde may act as a direct sensitizer or induce cross-sensitization in predisposed individuals (Inderbinen et al., 2022).
- Synthetic Dyes: Para-phenylenediamine (PPD), a component in many hair dyes, is a potent allergen that can provoke severe localized dermatitis and, occasionally, systemic reactions. Diagnosis is typically confirmed via targeted patch testing (Dalamaga et al., 2024).
- Trace Metals: Nickel, cobalt, and chromium—often present as impurities in pigments, applicators, or packaging—can provoke allergic reactions, especially in sensitized individuals. Nickel allergy remains one of the most common contact allergies globally (Ghosh et al., 2022).

#### 3.5.3 Diagnostic challenges

Diagnosis of cosmetic-related allergies is frequently complicated by several factors:

- Cross-reactivity: Chemical similarities between cosmetic allergens and compounds in foods or the environment can trigger reactions in sensitized individuals. For instance, those allergic to balsam of Peru may also react to cinnamon or citrus oils (Sakali et al., 2021).
- Poly-allergenicity: Cosmetics often contain multiple potential sensitizers, making it difficult to isolate the exact trigger, especially when using multiple products simultaneously (Koshko et al., 2022).
- Delayed Reaction Onset: ACD symptoms may not appear until 24–72 h post-exposure, complicating retrospective identification of the causative agent. Patch testing remains the diagnostic gold standard. Comprehensive panels like the TRUE Test or customized tests with patient-provided products are typically employed (Wu et al., 2023).

#### 3.5.4 Management and prevention

- Avoidance: Once allergens are identified, strict avoidance is paramount. Patients should be educated on ingredient label interpretation and guided toward suitable alternatives devoid of their specific allergens (Pistollato et al., 2021).
- Product Reformulation: Advances in cosmetic science have led to hypoallergenic formulations that minimize sensitizer use. While these reduce overall risk, no product can be universally non-allergenic (Ajayi et al., 2024).
- Regulatory Oversight: Regulatory frameworks like those enforced by the EU and FDA mandate ingredient disclosure and allergen labeling. However, inconsistencies in enforcement and scope remain barriers to comprehensive consumer protection (Debora et al., 2025).
- Barrier Support: Restoring the skin’s natural defense mechanisms is key. Emollients containing ceramides, hyaluronic acid, or occlusives support skin barrier repair and reduce vulnerability to irritants and allergens (Park and Hong, 2024b).

In conclusion, cosmetic-induced allergic reactions, especially ACD, pose significant dermatological challenges driven by both immune and non-immune mechanisms. A proactive approach emphasizing precise diagnosis, allergen avoidance, product innovation, and improved regulatory enforcement is essential to enhance safety and reduce the burden of allergic diseases associated with cosmetic use (Chen et al., 2022).

### 3.6 Skin disorders

Cosmetic-induced skin disorders are widespread, particularly among individuals with sensitive skin or pre-existing conditions such as eczema, rosacea, and psoriasis. These individuals often have compromised skin barriers, making them more susceptible to irritation and allergic reactions from various cosmetic ingredients. Contact dermatitis, one of the most frequent adverse reactions, results from direct exposure to irritants or allergens (Clouston et al., 2022). It can be categorized into irritant contact dermatitis (ICD) and allergic contact dermatitis (ACD), both of which cause redness, swelling, and discomfort. People with compromised immune systems or a history of eczema are particularly vulnerable to these reactions.

Recent research suggests that certain endocrine-disrupting chemicals (EDCs) found in personal care products may also contribute to inflammatory skin conditions. A longitudinal panel study that involved 18 boys aged 3–7 with diagnosed atopic dermatitis found that elevated urinary levels of mono-n-butyl phthalate (MnBP) and bisphenol A glucuronide (BPAG) were significantly associated with worsened atopic dermatitis (AD) on both the same day and the following day (Kim et al., 2017). Although the study was limited in its small sample size and its inclusion of only boys, it employed a robust time series design using 460 pooled urine samples and daily symptom diaries, while controlling for temperature and humidity. These findings reinforce that phthalates and BPA have inflammatory effects; hence, young children’s exposure through personal care products and packaging should be limited.

Another prospective birth cohort study involving 413 Korean mother-infant pairs found that higher maternal urinary concentrations Di(2-ethylhexyl) phthalate (DEHP) metabolites were associated with significantly increased risk of AD in infants at 6 months of age, especially among girls (Lee et al., 2021). BPA did not show a strong independent association in single-pollutant models, but it contributed to increased AD risk when considered as part of a chemical mixture. The study’s use of repeated urine sampling and Bayesian Kernel Machine Regression (BKMR) strengthened its ability to evaluate interactions between exposures. These results suggest that pregnant women should limit their exposure to products that contain phthalates and BPA to reduce the risk of AD in their babies.

Long-term exposure to certain cosmetic ingredients has been linked to more persistent skin issues, such as perioral dermatitis (a facial rash around the mouth and nose) and acneiform eruptions, which resemble acne but are often triggered by specific cosmetic products like heavy moisturizers or topical steroids (Goel et al., 2024). A case-control study found that urinary BPA concentrations were significantly higher in adults with acne (mean: 7.94 μg/g creatinine) compared to healthy controls (mean: 5.62 μg/g) (Kaya Ozden and Karadag, 2021). Higher BPA levels were also correlated with increased acne severity and earlier onset. Frequent use of products with high alcohol content or astringents can exacerbate dry skin, leading to further complications like irritation and peeling (Preller et al., 2024). The use of heavy or occlusive ingredients in skincare can also block pores and contribute to the development of cystic acne, especially in those predisposed to the condition. Over time, the cumulative effect of exposure to harsh ingredients can worsen the skin’s condition, leading to chronic inflammatory disorders that may require medical intervention. Furthermore, in one analysis, 44% of creams exhibited microbial contamination, surpassing EU safety thresholds 1 month after opening, suggesting poor preservative control and a heightened risk of skin infections (Irfan et al., 2022).

Given these risks, it is increasingly important to prioritize dermatologically tested formulations, especially for those with sensitive or reactive skin. Transparent ingredient lists are crucial in helping consumers identify known irritants or allergens. Dermatologists often recommend patch testing new products to minimize the risk of adverse reactions. Formulations free of parabens, artificial fragrances, sulfates, and other common irritants are essential for maintaining skin health and avoiding unnecessary flare-ups of existing conditions. Furthermore, consumers should be aware that even seemingly “gentle” products can trigger irritation if used inappropriately or in combination with other potent ingredients. Therefore, mindful product selection and a more cautious approach to skincare can significantly reduce the likelihood of cosmetic-induced skin disorders (CDC et al., 2019).

### 3.7 Neurological effects

Cosmetic products containing synthetic fragrances and volatile organic compounds (VOCs) have increasingly come under scrutiny for their potential neurotoxic effects. Neurological outcomes associated with such exposure span a continuum from acute symptoms such as headaches, migraines, and mood fluctuation to longer-term impacts involving cognitive dysfunction and emotional dysregulation. Although the exact molecular mechanisms remain incompletely understood, growing evidence suggests that certain VOCs can traverse the blood-brain barrier (BBB), a selective membrane that protects the central nervous system from harmful exogenous substances. Once within the brain, these compounds may disrupt neuronal communication, alter neurotransmitter dynamics, and induce oxidative stress, ultimately contributing to neuroinflammation and cellular injury (Mukherjee Das et al., 2022).

Acute exposure to elevated concentrations of synthetic fragrances or VOCs, particularly in confined or inadequately ventilated environments, has been correlated with immediate symptoms including vertigo, nausea, and sensory hypersensitivity. These effects are thought to arise from the stimulation of the olfactory and trigeminal nerve pathways, which are intricately connected to brain regions regulating emotion, pain, and autonomic function. In individuals with underlying vulnerabilities such as multiple chemical sensitivity (MCS) or chronic migraine syndromes, such exposures may precipitate severe exacerbations, often necessitating medical intervention and long-term symptom management (Attard et al., 2022).

Prolonged or chronic exposure to neuroactive VOCs presents a more covert threat, with potential implications for mood disorders, cognitive impairment, and even neurodegenerative conditions. Research has implicated VOCs in the dysregulation of key processes such as synaptic plasticity and neurogenesis, both of which are essential for learning, memory, and adaptive behavior. Persistent oxidative stress and inflammation triggered by VOC exposure may undermine neural integrity, resulting in behavioral disturbances, anxiety, and depressive symptoms (Vale et al., 2022). Moreover, chronic inhalation of these compounds has been shown to elevate cortisol levels and disrupt the hypothalamic-pituitary-adrenal (HPA) axis, further compromising emotional regulation and increasing the risk for affective disorders (Mukherjee Das et al., 2022). Prenatal exposure to BPA has been associated with neurodevelopmental disorders such as autism, ADHD, and learning deficits, likely due to its interference with brain and endocrine development (Chen et al., 2022). These early exposures may also impair organogenesis in systems such as the immune, reproductive, and nervous systems, with consequences that persist into adulthood.

The neurobehavioral risks associated with cosmetic VOC exposure necessitate a multidisciplinary investigative approach. Current evidence remains fragmented, and a comprehensive understanding of the dose-response relationship, susceptibility thresholds, and critical periods of exposure is lacking. Individual factors such as genetic polymorphisms in detoxification enzymes, pre-existing neurological or psychiatric conditions, and cumulative chemical exposures may significantly modulate vulnerability. Advances in neuroimaging techniques (e.g., functional MRI, PET scans) and the identification of neurotoxic biomarkers hold promise in elucidating the structural and functional brain changes attributable to cosmetic-related VOC exposure (Smith and Hotchkiss, 2023).

In conclusion, while the neurological effects of synthetic fragrances and VOCs in cosmetics are still being delineated, available evidence points to their potential role in both acute neurophysiological responses and long-term neurobehavioral dysfunction. The convergence of toxicological, neurological, and environmental research is essential to delineate causal pathways and to inform safer cosmetic formulation standards, especially for individuals with heightened neurological sensitivity.

### 3.8 Common cosmetic contaminants

Table 1 offers a detailed synthesis of prevalent chemical contaminants found in a wide range of cosmetic products, summarizing their common uses, typical exposure levels, associated health effects, existing regulatory limits, and the most vulnerable population groups. Substances such as parabens, phthalates, formaldehyde and its releasers, heavy metals (including lead and cadmium), triclosan, volatile organic compounds (VOCs), and UV filters like oxybenzone are frequently detected in everyday cosmetic items such as lotions, shampoos, sunscreens, deodorants, nail products, and makeup. These compounds are often added intentionally for functions like preservation, fragrance stabilization, or UV protection, while others, such as heavy metals, appear as unintentional contaminants due to raw material impurities or manufacturing processes.

Despite the often-low concentrations at which these chemicals are present, ranging from parts per million to percentages, their health implications can be significant, especially with chronic use and simultaneous exposure to multiple products. For instance, parabens and phthalates are recognized endocrine disruptors with links to reproductive toxicity and developmental issues. Formaldehyde, classified as a Group 1 carcinogen by the International Agency for Research on Cancer (IARC), is known to cause respiratory irritation and skin sensitization. Heavy metals like lead have no known safe exposure level, particularly for children, and have been associated with neurodevelopmental delays and carcinogenicity. Triclosan has been linked to antimicrobial resistance and hormonal disruption, while certain UV filters such as oxybenzone have shown estrogenic activity and potential environmental toxicity.

The table also highlights notable disparities in regulatory frameworks. While the European Union has established strict bans or concentration limits for many of these substances, the United States often permits their use with minimal restrictions, relying instead on industry self-regulation. This variation underscores a regulatory gap that may lead to increased consumer exposure in less strictly regulated markets. Vulnerable populations such as pregnant women, children, individuals with pre-existing hormonal conditions, and occupationally exposed groups like hairdressers are particularly susceptible to the harmful effects of these chemicals. Overall, the information presented in the table calls attention to the pressing need for comprehensive toxicological assessments, harmonized international regulations, and increased transparency in cosmetic product labeling. It also highlights the importance of raising public awareness about the potential health risks associated with long-term and cumulative exposure to chemical contaminants in personal care products.

### 3.9 Regulatory frameworks for cosmetic products

Regulatory frameworks for cosmetics may vary across regions, affecting how products are evaluated, labeled, and monitored. Table 2 compares key regulatory components across the United States, European Union, Canada, and ASEAN, highlighting differences in premarket requirements, safety assessments, ingredient restrictions, labeling, and enforcement mechanisms.

**TABLE 2:** Comparative overview of cosmetic regulatory frameworks.

Regulatory aspect	United States (FDA)	EU (Reg. 1223/2009)	Canada (F&DA)	ASEAN (AHCRS)
Premarket Notification	Mandatory under Modernization of Cosmetics Regulation Act (MoCRA)	Mandatory via Cosmetic Products Notification Portal (CPNP)	Mandatory (Cosmetic Notification Form)	Mandatory product notification to authority in each Member State where marketed
Premarket Safety Assessment	Not required; manufacturer responsible for maintaining substantiation of safety for each product.	Mandatory safety assessment and Cosmetic Product Safety Report (CPSR)	Not required, but manufacturer must ensure safety.	Required: Product Information File (PIF) with safety assessment by qualified Safety Assessor
Prohibited/Restricted Ingredients	Few (e.g., mercury, chloroform, vinyl chloride)	>1400 substances banned; positive lists for UV filters, preservatives, colorants	Dozens banned/restricted (Cosmetic Ingredient Hotlist)	Negative list (banned), Restricted list (limited use), Positive lists (UV filters, preservatives, colorants)
Color Additive Approval Required	Yes (except for coal-tar hair dyes)	Yes, and must be from approved batches.	Yes (must be approved for specific use).	Yes, positive list of permitted colorants must be followed.
Nanomaterial Regulations	No explicit nanomaterial labeling rule yet, but MoCRA enables FDA to regulate ingredient disclosure and may include nanomaterials.	Must be notified and labeled [nano]; special approval for new uses.	No specific rules on disclosure or assessment but industry is advised to disclose them voluntarily.	No specific labeling required for nano; but safety evaluation is expected.
Animal Testing Status	Permitted (not banned federally, some state bans)	Fully banned, including marketing of animal-tested products.	Banned federally (as of 2023).	No regional ban: individual ASEAN countries regulate independently; most have no ban in place.
Labeling Requirements	Basic INCI labeling under FPLA; no ingredient label needed for professional use	Extensive INCI labeling, batch no., usage instructions, PAO, warnings	INCI labeling, bilingual (English/French), CPLA compliant	Full INCI ingredient listing: clear labeling on packaging required (e.g., expiry date, batch number)
GMP Requirements	Mandatory: FDA is required to propose and enforce GMP regulations under MoCRA.	Mandatory (ISO 22716)	Expected to follow GMP but not mandated by law	Mandatory compliance with ASEAN Cosmetic GMP Guidelines
Enforcement Authority	FDA (post-market); collaborates with U.S. Customs	National competent authorities in each Member State	Health Canada	Each country’s national regulatory authority + ASEAN Cosmetic Committee
Recall Authority	Yes, FDA has authority to mandate recalls of unsafe cosmetics under MoCRA.	Yes (mandatory recall powers)	Yes (mandatory recall powers)	Yes (each country can enforce recalls; post-market surveillance emphasized).

Sources: FDA (Modernization of Cosmetics Regulation, 2024), European Commission (Legislation - European Commission, 2024), Health Canada (Cosmetic Regulations, 2024), ASEAN (ASEAN, 2024).

## 4 Limitations and future research directions

Despite growing evidence on the health risks of cosmetic and personal care products, several limitations hinder a conclusive understanding. The summary of evidence included in this study is presented in Table 3, alongside study population, the methodological strengths and limitations. Most existing studies are observational, limiting causal inference due to potential confounding and exposure misclassification. Long-term effects of chronic, low-dose, and cumulative exposure to multiple cosmetic chemicals remain underexplored, particularly in real-world settings where mixtures of substances are used daily. Vulnerable populations, including pregnant women, infants, and individuals with underlying sensitivities, are often underrepresented in research, despite their increased susceptibility. There is also a lack of standardized biomarkers and biomonitoring data specific to cosmetic-related compounds, making it difficult to assess internal exposure levels and biological responses. Regulatory inconsistencies across countries further complicate risk evaluation, with some harmful chemicals still permitted in certain markets. To address these gaps, future research should focus on longitudinal studies, advanced exposure assessment tools, mechanistic investigations using ‘omics’ technologies, and randomized controlled trials. A more harmonized, multidisciplinary approach is essential to inform safer cosmetic formulations and effective public health policies.

**TABLE 3:** Summary of key studies on cosmetic ingredients, exposure, and health effects.

Study	Study type	Population studied	Targeted Chemical(s)	Health outcomes	Strength of the study	Limitation of the study
Axelstad et al. (2011)	*In vivo* animal study	Pregnant Wistar rats and offspring	Octyl Methoxycinnamate (OMC)	• OMC exposure significantly reduced maternal thyroxine (T4) levels • Male offspring showed reduced testosterone, sperm count, and prostate and testis weights • Female offspring had reduced motor activity. Enhanced spatial learning in some males	Use of multiple doses Followed offspring into adulthood.	Animal-only data
Gomez et al. (2005)	*In vitro* study using ERα and ERβ reporter cell lines	Human HeLa-derived cell lines expressing estrogen receptors	Parabens, UV screens, musk fragrances	• Butyl- and propylparaben strongly activated both ERα and ERβ, indicating significant estrogenic activity • OMC, 4-MBC, OD-PABA, and HMS activated ERα only, suggesting selective tissue effects • Musk ketone and galaxolide weakly activated Erα	Used human-derived cell lines specific for ERα and Erβ Results were benchmarked against 17β-estradiol.	As an *in vitro* assay, it does not replicate metabolism, absorption, or systemic effects in living organisms.
Janjua et al. (2004)	Single-blinded controlled human exposure study	32 healthy adults (15 males, 17 postmenopausal females)	Benzophenone-3 (BP-3), OMC, 4-MBC	• All three UV filters were systemically absorbed and excreted in urine • No significant hormonal effects observed (FSH, LH, estradiol, testosterone, inhibin B)	Studied systemic absorption.	Short study duration (1 week) limits assessment of long-term effects.
Joensen et al. (2018)	Human biomonitoring with genotype-based subgroup analysis	195 healthy young Danish (65 *FLG*-mutant, 130 non-carriers)	BPA, parabens (MeP, EtP, nPrP, BuP), UV filters (BP-1, BP-3, 4-HBP), phenols	• In *FLG*-mutant carriers, higher urinary BPA and UV filters were linked to increased testosterone/estradiol, decreased FSH, and decreased sperm motility • No effects were seen in non-carriers • Parabens showed no associations with hormonal or reproductive markers	Included genotype stratification.	Used only single spot urine and semen samples per participant, limiting temporal resolution.
Kaya Ozden and Karadag (2021)	Prospective human case-control study	101 adults (18–25 years; 51 acne patients, 50 controls)	BPA, BPA-glucuronide	• BPA was significantly higher in acne group (7.94 vs. 5.62 μg/g) • BPA concentration positively correlated with acne severity and earlier age of onset.	Controlled for confounders such as BMI, diet, and age	Did not measure hormone levels directly. BPA exposure history was self-reported.
Kim et al. (2017)	Longitudinal time-series panel study	18 boys (3–7 years) with atopic dermatitis	Phthalates (MnBP, MEHHP, MEOHP), BPA	• Higher urinary MnBP and BPAG levels were linked to increased AD symptoms the same day and next day	Included repeated urine sampling (460 pooled samples), seasonal tracking, and validated diary-based health records.	Only boys were included. The sample size was small (N = 18).
Lee et al. (2021)	Prospective birth cohort	413 Korean mother–infant pairs	BPA, phthalates (MEHHP, MEOHP, MnBP)	• Late pregnancy phthalate exposure linked to increased risk of atopic dermatitis in 6-month-old infants, especially girls • BPA showed effect only in chemical mixture models	Used repeated exposure measurements (early and late pregnancy). Large and well-characterized birth cohort	AD was parent-reported without clinical confirmation. Did not measure infant exposure postnatally.
Rees Clayton et al. (2010)	Cross-sectional population-based biomonitoring study	3,728 U.S. participants ≥6 years old	Bisphenol A (BPA), triclosan	• Higher urinary BPA levels were associated with elevated CMV antibody levels in adults • Higher triclosan levels were linked to increased odds of allergy or hay fever in children		
Husøy et al. (2019)	Cross-sectional human biomonitoring study (24-h urine)	144 Norwegian adults (100 females, 44 males) aged 24–72	Parabens (MEPA, ETPA, PRPA, BUPA), bisphenols (BPA, BPS, BPF), UV filter (benzophenone-3), antimicrobials (TCS, TCC), multiple phthalates	• Methyl- and ethyl-parabens were more frequently detected among female participants • Benzophenone-3 and triclosan were found in over 90% of urine samples • Phthalate levels were generally higher in males than in females • The study identified associations between exposure to these chemicals and hormone-related toxicity and metabolic disruption • Health risks were linked to frequent use of lip balm, sunscreen, shaving cream, and toothpaste	Used full 24-h urine samples instead of spot urine, improving exposure accuracy for short half-life chemicals. The study included detailed logs of food and personal care product use. Large sample size	Self-reported data may be prone to underreporting.
Irfan et al. (2022)	*In vitro* chemical analysis and toxicological risk assessment	Nine commercially available fairness creams from local and international brands	Heavy metals (Cd, Cr, Ni, Pb, Zn, Hg), hydroquinone, and microbial contamination	• Mercury and zinc were detected at very high levels, with mercury exceeding regulatory safety thresholds and linked to elevated hazard quotient (HQ) and lifetime cancer risk (LCR) • Lead, cadmium, nickel, and chromium were also found and associated with genotoxicity, nephrotoxicity, allergic reactions, and carcinogenicity • Hydroquinone was present in 89% of creams, often unlabeled, at concentrations exceeding international safety limits, posing risks such as ochronosis, irritation, and carcinogenicity • Microbial contamination was observed in 44% of samples, indicating poor preservative control and an increased risk of skin infections	Used validated instrumentation. Risk was assessed using SED, MoS, HQ, HI, and LCR metrics.	No *in vivo* human testing Limited to only nine samples, and actual consumer use patterns were not simulated.
Lim et al. (2018)	Laboratory-based quantitative risk assessment	200 cosmetic products from the Korean market	14 heavy metals: Al, Cr^3+^/Cr^6+^, Mn, Fe, Co, Ni, Cu, Zn, As, Pb, Hg, Cd, Sb, Ti	• Most products showed margins of safety (MOS) > 100 and hazard indices (HI) < 1, suggesting negligible systemic toxicity • Lifetime cancer risk (LCR) values for Cr^6+^, Ni, As, Pb, and Cd were below the regulatory threshold of 10^−6^ • Some lip and eye products exceeded acceptable daily intake for Cr^6+^, Mn, and Pb among heavy users	Extensive and robust sample size (200 products)	Assumed 100% ingestion or dermal absorption in some scenarios. Did not explore cumulative effects of multiple metals or long-term real-world use.
Salles et al. (2023)	Laboratory-based chemical analysis	95 costume cosmetics	Al, As, Ba, Cd, Co, Cr, Cu, Ni, Pb, Sb, Sn, Sr	• All products contained multiple toxic elements, with arsenic contributing to ∼90% of total cancer risk • In children, lifetime cancer risk from ingestion exceeded acceptable thresholds (up to 10^−5^) • In adults, dermal exposure resulted in elevated cancer risk (up to 10^−3^)	Calculated both dermal and ingestion exposure, including realistic age- and sex-specific variables.	Did not assess mixture effects with other cosmetics or co-exposure from diet/environment.
Voica et al. (2023)	Laboratory-based toxicological risk assessment	14 facial cosmetics (10 lipsticks, 4 eye shadows)	Cr, Co, Ni, Cu, As, Pb, Hg, Cd	• Lead, chromium, and mercury exceeded international safety thresholds in several lipstick and eye shadow samples • Lead levels reached 27.0 mg/kg in lipstick and 40.9 mg/kg in eye shadow, while chromium ranged up to 149 mg/kg • These metals are linked to systemic toxicity, dermatitis, neurotoxicity, reproductive harm, and carcinogenicity	Modeled both 50% and 100% bio accessibility.	Did not assess real-world usage frequency or actual dermal absorption. Limited sample size (N = 14)

## 5 Conclusion

In light of the mounting evidence, the cosmetic industry stands at a critical crossroads between innovation and accountability. Ensuring product safety without sacrificing efficacy requires an integrated strategy involving rigorous ingredient screening, investment in green chemistry, and adherence to scientifically validated safety thresholds. Regulatory bodies should establish and enforce clear limits on toxic ingredients, and regulatory frameworks must evolve to include mandatory pre-market safety assessments. Continual post-market surveillance and routine testing are needed to ensure product safety. Current evaluations often overlook the real-world impacts of chronic, low-dose exposure to chemical mixtures. More longitudinal human studies, especially in vulnerable populations such as children, pregnant women, and individuals with genetic susceptibilities are needed. Safety margins should be reassessed for chemicals that are systemically absorbed, such as UV filters. Equally important is fostering a culture of transparency, where consumers are empowered through clear labeling and access to ingredient safety information. Cosmetic manufacturers should actively avoid combining multiple weak estrogenic compounds, especially in products intended for long-term dermal application. Educational campaigns targeting both consumers and healthcare providers can facilitate better recognition of adverse reactions and promote safer product choices. Ultimately, safeguarding public health demands a coordinated effort among scientific communities, regulatory authorities, manufacturers, and consumers. By embracing a proactive, evidence-based approach, the cosmetics sector can transition toward more sustainable and health-conscious practices, ensuring that beauty does not come at the cost of well-being.
