#  Correction: The impact of perfumes and cosmetic products on human health: a narrative review

**DOI:** 10.3389/ftox.2025.1713750

**Published:** 2025-12-16

**Authors:** Sharifa Alblooshi

**Affiliations:** College of Natural and Health Sciences, Zayed University, Dubai, United Arab Emirates

**Keywords:** perfumes, cosmetics, health risks, synthetic chemicals, regulatory guidelines, endocrine disruption, heavy metals, consumer safety

In the **Abstract**, the sentence was incorrectly written with incorrect grammar as:

“A comprehensive review of the literature that was published between 2005 and 2024 was conducted…”

This has been corrected to read:

“A comprehensive review of literature published between 2005 and 2025 was conducted…”

The text was incorrectly written with incorrect hyphenation as:

“neurological wellbeing”

A correction has been made to the **Introduction**, paragraph 6.

“neurological well-being”

The paragraph repeats the same idea discussed in previous paragraphs and is written as:

“Cosmetics have become an integral part of modern life, used daily by millions of people to enhance appearance, boost confidence, and maintain personal hygiene (**Gabriella, 2023**). From skincare to makeup and haircare products, the cosmetic industry offers an extensive variety of formulations designed to cater to diverse needs and preferences. While these products provide undeniable benefits, their widespread use has also raised concerns about potential health risks associated with prolonged exposure to certain chemical ingredients (**Naidu et al., 2021**).”

A correction has been made to the **Introduction**, paragraph 7:

The paragraph has been removed.

The text was incorrectly written as: “…and effects of heavy metals.” A correction has been made to the Introduction, paragraph 10:

““…and the effects of heavy metals.”

The text was incorrectly written with incorrect year range as:

“Studies that were published in the years between 2005 and 2024 were included to provide a well rounded and comprehensive perspective on the topic.”

A correction has been made to **Section 2**, paragraph 1:

“Studies that were published in the years between 2005 and 2025 were included to provide a well rounded and comprehensive perspective on the topic.”

The text was incorrectly written as:

“Studies with limited relevance or non-generalizable results (e.g., small sample sizes, non-human studies).”

A correction has been made to **Section 2**, *sub-section 2.2*:

“Studies with limited relevance or non-generalizable results (e.g., small sample sizes).”

The text was written incorrectly with incorrect punctuation as:

“See **Table 1**, for health risks associated with …”

A correction has been made to **Section 3**, *sub-section 3.1*, paragraph 1.

“See **Table 1** for health risks associated with …”

The text was written incorrectly with incorrect label as:

“Musk ketone and galaxolide weakly activated Erα; …”

A correction has been made to **Section 3**, *sub-section 3.2*, paragraph 3.

“Musk ketone and galaxolide weakly activated ERα; …”

The text incorrectly included the citation “(**Boos, 2023**),” which is unrelated to cosmetic aerosol exposures.

“Aerosolized cosmetics, including spray deodorants, dry shampoos, and setting sprays, contribute an additional hazard by emitting fine and ultrafine particulate matter (PM2.5 and PM0.1), which can penetrate deep into the lungs (**Boos, 2023**). These particles bypass the upper airway’s filtration mechanisms and deposit in the alveolar regions, where they induce oxidative stress and trigger inflammatory cascades. Persistent exposure to such particles is associated with structural changes in lung tissue, impaired gas exchange, and a heightened risk of respiratory infections. Vulnerable groups such as children whose lungs are still maturing and elderly individuals with pre-existing conditions like asthma or COPD are especially susceptible to these effects, with exposure linked to increased frequency and severity of exacerbations, hospital visits, and long-term morbidity (**Attard et al., 2022; Ullah et al., 2023**).”

A correction has been made to **Section 3**, *sub-section 3.3*, paragraph 3:

“Persistent exposure to aerosolized cosmetics, including spray deodorants, dry shampoos, and setting sprays is associated with structural changes in lung tissue, impaired gas exchange, and a heightened risk of respiratory infections. Vulnerable groups such as children whose lungs are still maturing and elderly individuals with pre-existing conditions like asthma or COPD are especially susceptible to these effects, with exposure linked to increased frequency and severity of exacerbations, hospital visits, and long-term morbidity (**Attard et al., 2022; Ullah et al., 2023**).”

The text was written incorrectly as:

“bio-monitoring data”

A correction has been made to **Section 4**, paragraph 1:

“biomonitoring data”

The text was written incorrectly with incomplete sentence as:

“Despite growing evidence on the health risks of cosmetic and personal care products, several limitations hinder a conclusive understanding. Limitations and future research directions section, The summary of evidence included in this study is presented in **Table 3**, alongside study population, the methodological strengths and limitations.”

A correction has been made to **Section 4**, paragraph 1:

“Despite growing evidence on the health risks of cosmetic and personal care products, several limitations hinder a conclusive understanding. The summary of evidence included in this study is presented in **Table 3**, alongside study population, the methodological strengths and limitations.”

The text was incorrectly written with incorrect hyphenation as:

“…. at the cost of wellbeing”

A correction has been made to **Section 5**, paragraph 1:

“…at the cost of well-being”

The published article, the citations “**Gabriella, 2023**” and “**Naidu et al., 2021**” in **Introduction**, paragraph 7 were removed because the paragraph in which they were cited was removed.

The sentence originally read:

Cosmetics have become an integral part of modern life, used daily by millions of people to enhance appearance, boost confidence, and maintain personal hygiene (**Gabriella, 2023**). From skincare to makeup and haircare products, the cosmetic industry offers an extensive variety of formulations designed to cater to diverse needs and preferences. While these products provide undeniable benefits, their widespread use has also raised concerns about potential health risks associated with prolonged exposure to certain chemical ingredients (**Naidu et al., 2021**).”

This paragraph has been removed. The references **Gabriella (2023)** and **Naidu et al. (2021)** have also been removed from the reference list.

In the published article, the citation “**Boos, 2023**” was erroneously included in **Section 3**, *Sub-section 3.3*, paragraph 3. This reference is not relevant to cosmetic aerosol exposures.

The sentence originally read:

“Aerosolized cosmetics, including spray deodorants, dry shampoos, and setting sprays, contribute an additional hazard by emitting fine and ultrafine particulate matter (PM2.5 and PM0.1), which can penetrate deep into the lungs (**Boos, 2023**). These particles bypass the upper airway’s filtration mechanisms and deposit in the alveolar regions, where they induce oxidative stress and trigger inflammatory cascades. Persistent exposure to such particles is associated with structural changes in lung tissue, impaired gas exchange, and a heightened risk of respiratory infections. Vulnerable groups such as children whose lungs are still maturing and elderly individuals with pre-existing conditions like asthma or COPD are especially susceptible to these effects, with exposure linked to increased frequency and severity of exacerbations, hospital visits, and long-term morbidity (**Attard et al., 2022; Ullah et al., 2023**).”

This has been corrected to:

“Persistent exposure to aerosolized cosmetics, including spray deodorants, dry shampoos, and setting sprays is associated with structural changes in lung tissue, impaired gas exchange, and a heightened risk of respiratory infections. Vulnerable groups such as children whose lungs are still maturing and elderly individuals with pre-existing conditions like asthma or COPD are especially susceptible to these effects, with exposure linked to increased frequency and severity of exacerbations, hospital visits, and long-term morbidity (**Attard et al., 2022; Ullah et al., 2023**).”

The reference **Boos (2023)** has also been removed from the reference list.

The original article has been updated.

